# Students’ Performance in Face-to-Face, Online, and Hybrid Methods of Teaching and Assessment in Anatomy

**DOI:** 10.3390/ijerph192013318

**Published:** 2022-10-15

**Authors:** Halima Albalushi, Mohamed Al Mushaiqri, Srinivasa Rao Sirasanagandla, Srijit Das

**Affiliations:** Department of Human & Clinical Anatomy, College of Medicine & Health Sciences, Sultan Qaboos University, Muscat 123, Oman

**Keywords:** anatomy, COVID-19, assessment, medical education, online, face-to-face, hybrid, teaching

## Abstract

In recent times, online teaching and assessment have provided a great opportunity to explore better methods in medical education. There are inconsistent views concerning the effectiveness of online assessment. Hence, the present study aimed to evaluate online teaching and assessment methods in relation to face-to-face methods by comparing students’ performances. The students’ assessment results in two basic anatomy courses, which are part of the Doctor of Medicine and Biomedical Sciences programs at Sultan Qaboos University, were analysed. We compared the students’ mean scores and coefficient of variance in the multiple-choice written exams and the objective structured practical exams during the spring semesters of 2019, 2020, and 2021, containing face-to-face teaching and exams, partial online teaching and online exams, and online teaching and both proctored online and face-to-face exams, respectively. The sudden transition to online teaching and assessment halfway through the semester resulted in higher means and a lower coefficient of variance among students’ scores in both theory and practical exams. However, when the fully adopted online method of teaching and assessment was employed, the mean scores decreased, and the coefficient of variance increased to figures close to those witnessed before the pandemic, when teaching and assessment were face-to-face. This trend applied to both the Doctor of Medicine and Biomedical Sciences programs’ anatomy courses. The results indicate that online assessment of theoretical and practical anatomical knowledge is comparable to that of face-to-face assessment. However, proper planning and preparedness are mandatory to achieve the desired outcomes.

## 1. Introduction

The COVID-19 pandemic affected many countries all over the world. The outbreak started in the latter part of 2019 and was declared a pandemic by the World Health Organization (WHO) in 2020 [1]. All nations went into complete lockdown, as there was a need to stop the spread of infection and flatten the curve, which affected all aspects of life [2]. The pandemic resulted in many classes suspended in various medical institutions worldwide. Under such conditions, universities across the globe were affected by the pandemic and the sudden transition from face-to-face to online teaching. University curricula and teaching techniques changed along with other “remote” professional activities in this shifting environment [3]. Research studies have highlighted several opportunities for the development of distance education and the technical readiness for such during the COVID-19 pandemic [4].

Medical students had to face various social, economic, and cultural factors, which acted as barriers to their personal and academic lives, and the situation was more challenging for minority and underprivileged students [5]. Interestingly, a study showed that COVID-19 had a negative effect on medical students from high-income, middle-income, and low-income countries [5]. In order to address the challenges posed by COVID-19, the entire education sector saw a pedagogical shift in teaching towards e-learning [6].

During the pandemic, all courses and assessments were conducted online. Many institutions were not geared up for such a change in the method of teaching. Online teaching has played a major role in the education system of every profession. Nowadays, medical students always look for innovative teaching methods during online teaching [7]. During the pandemic, there was little time available for switching from regular physical sessions to online sessions, and the lecturers had the herculean task of looking for innovative online teaching and assessment methods. Online teaching was considered a time-consuming and intensive process because of the lack of infrastructure available [8]. It may be mentioned that no one was prepared for online teaching, and it came as an emergency mode of teaching. Online teaching and assessment also allowed us to explore better teaching methods and improve our medical education.

During the extraordinary circumstances of COVID-19, anatomy education was almost completely relayed using online resources and distance learning [9,10]. Various new methods of online instruction were introduced to support the online learning environment [11]. Online education could be delivered in the form of asynchronous distance education (ADE), where podcasts and recorded videos were used, or synchronous distance education (SDE), where virtual classrooms or live video conferences were used [12]. Currently, a blended form of delivery method, which is frequently called a “flipped classroom”, is used by many medical schools. In this scenario, students have the flexibility to attend both ADE and SDE [11]. In a recent meta-analysis of randomised clinical trials, it was clearly indicated that students preferred SDE to traditional education [12]. Hybrid instruction engages students’ learning by combining face-to-face (physical) and online (remote) learning opportunities. This method of learning offers a high level of flexibility [13]. Hybrid instruction has been reported to be helpful in improving the effectiveness of physiology teaching [14] and encouraging students to engage in active learning and problem solving in a pathophysiology course [15]. A hybrid flexible (HyFlex) teaching model refers to an instructional approach that combines face-to-face instruction with online synchronous or asynchronous content delivery. In the HyFlex model, students can choose to study either face-to-face or online, thus conferring greater chances of reaching out to students. In studies by Miller et al. (2013) and Shek et al. (2022), this model promoted students’ understanding of the subject objectives [16,17]. In another study, around 85% of the students were satisfied with this mode of teaching [18].

Learning design (LD) is defined as “the act of devising new practices, plans of activity, resources and tools aimed at achieving particular educational aims in a given situation” [19]. Developing an LD enables one to formally describe and procedurally encode educational activities [20]. It has been encouraged to engage with new developments around LD for better practices in contemporary healthcare education [20].

There are several concerns regarding medical students’ education including their immediate outcomes, work force requirements, and the available resources. The sudden change from face-to-face to online teaching also placed an additional burden on teachers to teach while measuring students’ achievement at regular intervals. One of the main objectives of any assessment is for the students to attain a specified standard before being labelled as ‘competent’ [21]. Online assessment results may benefit both students and teachers. It has been found that there was higher student achievement and promising staff perception, with improved technological skills, while implementing e-learning and online assessment during COVID-19 [22]. According to published studies, the integrity of assessment is a vital and challenging issue, especially as testing becomes more commonly distant from the usual classroom setting [23]. Online examinations can be proctored. Different ways include in-person testing (requiring students to be physically present at a testing session, which could be at the institution or administered by an approved proctor situated remote from the institution, depending on an honour system) or utilizing online real-time proctor services [23]. Furthermore, there is a need for an innovative assessment method for objective structured practical examination (OSPE) conduction in anatomy classes, as the traditional approach is associated with logistics and is time-consuming. There is a need to have new methods of conducting OSPE exams which test both comprehension and knowledge, unlike the old methods, which laid more emphasis on recall knowledge.

Although several studies looked at the effectiveness of online and offline teaching [24], there were differences in opinion among various researchers. Online assessment was found to stimulate students to be more responsible learners [25]. It was found that students liked online learning, as it offered well-structured learning resources and allowed them to study at home at their own speed and convenience [26,27]. Self-regulated and collaborative learning were related to learning achievements [27]. It was argued that there is a need to provide learning activities online that support and encourage collaborative learning, considering the advantages of this method for academic success [28]. Online collaboration was reported to be successful, especially when it is task related [28]. On the other hand, students liked face-to-face learning for specific reasons, which included acquiring motor skills and developing interpersonal relations [29]. Researchers have reported that achievement of the student learning outcomes was less efficient with online learning when compared to face-to-face learning [30,31]. Asghar et al. (2022) suggested more weight should be given to face-to-face learning considering the nature of vocational skills, which are practical based and require more hands-on experience [32].

Interestingly, there are a few research studies which did not find any significant differences between online and face-to-face learning [33,34]. A quasi-experimental study conducted on a master’s course showed that despite the sudden urgent change in the methodologies in teaching, students’ time performance did not decrease [3].

Considering the differences in opinion, there is a need for further studies to spell out the importance of online teaching and assessment in anatomy, and whether such can be employed, even after the end of the pandemic. There is also a need for an accepted online teaching method for anatomy that can be effectively used in the future. To the best of our knowledge, there is a paucity of studies exploring the effectiveness of online anatomy assessments compared to face-to-face assessments. Hence, the present study was conducted to assess students’ performance with regard to online and face-to-face assessments.

## 2. Materials and Methods

### 2.1. Study Design

A retrospective study to determine the effectiveness of online anatomy assessment compared with face-to-face assessment.

### 2.2. Ethics Statement

The Medical Ethics Research Committee at the College of Medicine and Health Sciences, Sultan Qaboos University approved the study.

### 2.3. Participants

This study included undergraduate MD (*n* = 465) and BMS students (*n* = 88) who attended introductory anatomy courses at the College of Medicine and Health Science (Table S1). We compared students’ performances in the theoretical and practical components of two introductory anatomy courses in three different semesters: spring 2019, spring 2020, and spring 2021. In these semesters, students were exposed to three different teaching and assessment methods. In all three semesters, the same instructors taught the anatomy subject. In the spring 2019 semester, students attended classes and exams face-to-face for the theoretical and practical parts of the course. In the spring of 2020, during the first half of the semester, students were exposed to face-to-face teaching methods, while in the second half of the semester, teaching and exams were conducted using the online teaching and assessment method. We named this method a hybrid method. In the spring 2021 semester, all course activities of teaching and assessment were conducted through the online method, but the exams and assessments were proctored (Table 1).

### 2.4. Setting

In the face-to-face teaching and assessment method, the theoretical activities, including lectures, tutorials, group activities, and exams, were conducted at designated venues in the College of Medicine and Health Sciences. When the COVID-19 situation imposed physical distancing and lockdown strategies, the online teaching and assessment method was followed, wherein students were provided with pre-recorded lectures. During this time, students could access all the teaching materials in the common teaching platform, i.e., Moodle. For the practical part during face-to-face teaching, anatomy demonstrations were scheduled weekly using the structured practical anatomy demonstration (SPRAD) approach. SPRAD is regarded as a standardized learning activity [35].

Briefly, students were divided into groups and spent two hours in the dissection hall with various learning materials, including cadavers, prosected specimens, plastic models, and radiological and microscopic images. From March 2020, the instructor taught all anatomy demonstration sessions with video recordings and recorded PowerPoint slide shows of the cadavers, plastic models, prosected specimens, histology slides, and radiology films.

### 2.5. Variables

We analysed the students’ assessment results in two introductory anatomy courses offered in the MD and BMS programs. Students’ performances on multiple-choice theory exams and objective structured practical exams (OSPE) during face-to-face teaching and assessment, partial online teaching and online assessment, and online teaching and proctored online assessment were compared to evaluate the effectiveness of online teaching and assessment methods over face-to-face teaching and assessment methods. A coefficient of variance (CV) was used to evaluate the variation in the students’ scores.

### 2.6. Statistical Analysis

Data were analysed using SPSS v.23. Descriptive statistics were used, and the data were presented as means ± standard deviation. The students’ performance scores in the theoretical and practical components of each course in the three semesters were analysed using the ANOVA test, followed by Tukey’s post hoc test when the ANOVA test showed significant differences. The level of significance was set at *p* < 0.05.

## 3. Results

This study aimed to compare student performance in basic anatomy courses of MD and BMS programs among three consecutive semesters, each with a different teaching and assessment method. The students’ average scores in the theory and practical assessments of the anatomy course in the spring 2019, spring 2020, and spring 2021 semesters are presented in Table 2 and Table 3.

### 3.1. Medical Students’ Results

**Figure 1:**
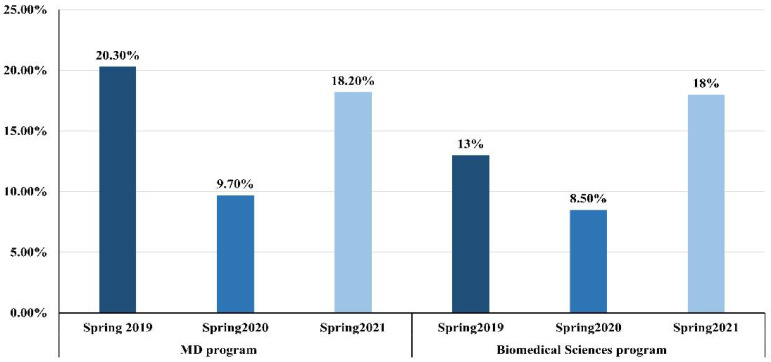
The co-efficient of variance calculated for the students’ scores in the theoretical component of basic anatomy course for MD and biomedical sciences programs in three semesters. CV = SD/Mean × 100.

For the theoretical assessment, the score in the spring 2020 semester (89.1 ± 8.6) was significantly higher than those in the spring 2019 (79 ± 16, *p* < 0.001) and spring 2021 (81.4 ± 14.8, *p* < 0.001) semesters in both the MD and BMS programs (Table 2). The variation among students’ scores is represented by the CV. The high average score in spring 2020 was accompanied by a reduction in the variation among students’ scores as represented by the CV value of 9.7%, which was lower than the value witnessed in spring 2019 (20.3%). However, the CV increased in spring 2021 to 18.2%, which was closer to the score observed in the spring 2019 semester when the whole course was taught and assessed with the face-to-face method (Figure 1).

**Figure 2:**
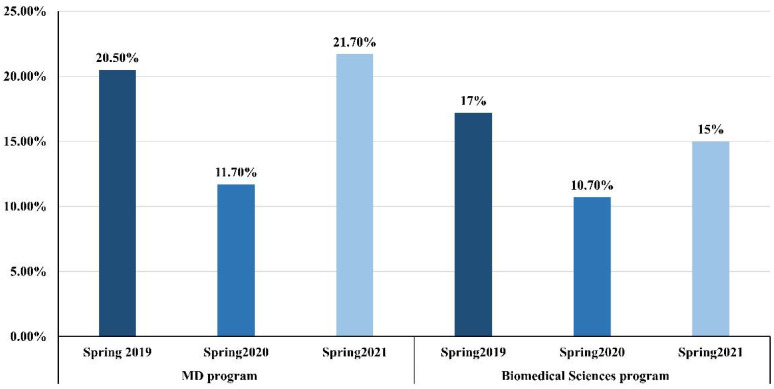
The co-efficient of variance calculated for the students’ scores in the practical component of basic anatomy courses for MD and biomedical sciences programs in three semesters. CV = SD/Mean × 100.

Similarly, the students’ scores on the practical assessment in the spring 2020 semester (85.8 ± 10) were significantly higher than those in the spring 2019 (77.4 ± 15.9, *p* < 0.001) and spring 2021 (78.7 ± 17.1, *p* < 0.001) semesters (Table 3). The CV of students’ scores among the three semesters was reduced in spring 2020 to 11.7% from 20.5% in spring 2019 but increased in spring 2021 to 21.7%, which was closer to the score observed in spring 2019 (Figure 2).

### 3.2. Biomedical Science Students’ Results

The average scores and CV of students’ scores in the theory and practical anatomy assessments of BMS students among the three semesters are presented in Table 2 and Table 3. For the theoretical assessment, the scores in the spring 2020 semester (89.4 ± 7.6) were significantly higher than those in the spring 2021 (80 ± 14.5, *p* = 0.008) and spring 2019 (84.8 ± 11) semesters. In a trend similar to that presented in the MD course results, the CV was reduced to 8.5% in spring 2020 from 13% in spring 2019, but in spring 2021 it increased to 18% (Figure 1).

The same trend was also witnessed among the practical assessment results. The score in spring 2020 (87.6 ± 9.4) was significantly higher than the score in spring 2019 (75.7 ± 13, *p* = 0.001). Similarly, in spring 2020, the CV was reduced to 10.7% from 17.2% in spring 2019. However, it increased to 15% in spring 2021 (Figure 2).

## 4. Discussion

Teaching and learning anatomy through the online method is always a challenge. According to researchers, a significant challenge is ensuring appropriate methods of examination in any online course [36]. Online assessments could also provide continuous and real-time feedback. Researchers have argued that a failure to modify the existing teaching and assessment methods could have a significant long-term impact on the educational trajectory and career progression of the younger generation throughout the world [37]. Assessments can measure any student’s performance, competence level, lacunae in learning, preparedness to progress, and entrustment of professional activities [38]. Assessment helps prepare students for the practice of medicine and to develop important professional values in later life [11]. Assessments as learning tools, such as portfolios or reflection exercises, may help students to be metacognitive and self-regulate their learning [38]. Anatomy, being a foundation subject in the initial part of the medical career, has a vital role in life-long learning. An extant search of the literature revealed few studies on the effectiveness of online methods of assessment in anatomy subjects. Hence, this prompted us to choose the basic “introduction to anatomy” course to explore this question.

Due to the lockdown during the pandemic, traditional OSPE inspection was impossible due to a lack of access to specimens and laboratory facilities. As a result, it was important to implement alternatives, such as online anatomy practical tests, to assist and assess student learning. Previously, online examinations in anatomy were conducted in which digital representations of the same materials utilized in the laboratory were used as question stems rather than actual specimens [39,40]. According to research on online anatomy spotting exams, there was no significant difference between the traditional OSPE approach and the online approach in terms of student performance [41,42]. Considering the epidemic, an online anatomy OSPE was therefore reasonable. The practical videos for online teaching were prepared using the cadaveric specimens and laboratory models that were generally used in the regular face-to-face teaching. In addition, in our institution, we used the structured practical material with defined tasks for students for practical training. Due to these two reasons, we assume that the students would have received similar training in both face-to-face and online practical delivery methods.

We analysed the students’ performances during the spring semesters in 2019, 2020, and 2021, wherein students were exposed to three different teaching and assessment methods, as mentioned earlier. Our study subjects included MD and BMS students who took the “introduction to human anatomy” course as part of their programs. This course is delivered during the spring semester of each year and carries four credit hours. In this course, all the basics in anatomy, including general anatomy, basic histology, and introduction to all systems of the human body are taught to the students.

In the spring 2020 semester, which witnessed the pandemic’s beginning, students’ mean scores in both theory and practical examinations were higher than in the pre-pandemic semester (spring 2019). At first thought, this may indicate a better performance. However, the high mean scores were accompanied by a lower CV, reflecting the online exams’ limited capacity to capture the variation in students’ anatomical knowledge. Considering the fact that students took the online exams during the spring 2020 semester from home and without proctoring, the high mean score may be attributed to students not adhering to academic integrity requirements.

Online assessment is time-consuming, requires considerable time for preparation, and lacks proper control of tests [43]. Online assessment may also need appropriate technology investment concerning hardware, software, and training [43]. Often, students residing in remote residential areas face the problem of internet speed while taking online examinations. The incidence of cheating also increases during online examinations, and strict guidelines must be imposed. Interestingly, search engine activity data on exam cheating collected in Spain revealed a significant increase in requests for information on exam cheating during online exams held during the COVID-19 pandemic [44]. Thus, academic integrity may be compromised. At times, there have been privacy issues, especially with the web cameras’ operation. In spring 2020, with the sudden transition to online teaching and assessment, faculty were not geared up with the online teaching and assessment methods and several hurdles were encountered.

The vagaries of the online method were a tremendous challenge for all of us. Becoming acquainted with new information technology methods was a hurdle for both students and teachers. In spring 2021, faculty and administrators were more geared up with all the necessary infrastructure needed for online teaching and assessment, and the results of the study showed that the students’ performance indicators in both theoretical and online assessment improved to figures close to those observed during the face-to-face method of teaching and assessment before the pandemic. This indicates that the online method of teaching and assessment with proper proctoring works well and is on par with the traditional method of teaching and assessment, as the results were within the expected ranges. Furthermore, this also shows the excellent preparedness of the faculty in the year 2021.

The results of the present study are further strengthened with findings from an earlier study, which showed that all the changes made during the COVID-19 pandemic showed learning effectiveness [3]. Another study showed that the network scale of most courses did not change significantly, giving evidence that the COVID-19 pandemic did not notably change the scale of course interaction [45]. Interestingly, even before the COVID-19 pandemic, a research study showed that online or blended learning for teaching clinical skills is equally effective and similar to traditional means, and it may even benefit the teaching of clinical skills in undergraduate nursing curriculum [46]. It is always important to develop such clinical skills in the profession.

There are several advantages to the online assessment method. It removes the hurdle of using traditional paper-based evaluation and the time needed for grading [47]. Online assessment is also reported to increase objectivity in grading, enables personalized evaluation, incurs less cost, motivates students, and promotes deep learning [48].

Our findings indicate that online assessments can be as good as face-to-face assessments. However, appropriate planning, faculty readiness, and appropriate technological means are required. Online courses may allow students to participate even from a distance. Moreover, with rapid mutations of the coronavirus, we never know when the pandemic may happen again. Hence, it is better to be equipped with online teaching and assessment methods.

The present study may have a few limitations. We evaluated the students’ performances with online and face-to-face methods of instruction during a sudden and unexpected campus lockdown caused by the COVID-19 pandemic. Admittedly, we evaluated the findings of the online versus face-to-face assessments under the prevailing circumstances, and the effectiveness may be limited by the fact that this was a single study with low sample size. Also, the difference between the types of assessment and teaching methodology used during the period of the study (prior and during the pandemic) could be a limitation. The main outcome measure of the study may not be a validated objective measure.

## 5. Conclusion

Assessment is an integral part of the medical curriculum. The sudden shift to online teaching at the start of the COVID-19 pandemic was accompanied by a mandatory shift to online assessment, which presented an excellent opportunity to investigate the effectiveness of online assessment for anatomy courses. Our findings indicate that the sudden shift to online assessment of anatomy courses with insufficient planning compromised the assessment through score inflation and reduced variation. However, with better planning and execution of online teaching and assessment later during the pandemic, online assessment of theoretical and practical anatomical knowledge was found to be as good as traditional face-to-face assessment in terms of average scores and variation among scores. Accordingly, anatomists should not always confine themselves to one traditional method of teaching and assessment and should always be open to innovative changes. Well-designed assessment programs with innovative methods can be beneficial.

## Figures and Tables

**Table 1:** Description of the teaching and assessment methods used during the three different semesters.

Semester	Teaching Methods	Assessment Methods
Spring 2019	Face-to-face	Face-to-face, proctored, on campus
Spring 2020	Face-to-face (first half of the semester) Emergency remote teaching (ERT) (Second half of the semester)	Online (not proctored)
Spring 2021	Online	Online (proctored) and face-to-face

**Table 2:** Students’ scores in the theoretical component of basic anatomy courses for MD and BMS programs in three different semesters. CV: Coefficient of variance is calculated as SD/Mean × 100.

Course	Semester (No. of Students)	Mean ± SD	Coefficient of Variance (%)
MD program basic anatomy Course	Spring 2019 (170)	79 ± 16	20.3
	Spring 2020 (166)	89.1 ± 8.6	9.7
	Spring 2021 (129)	81.4 ± 14.8	18.2
Biomedical Science program basic anatomy course	Spring 2019 (33)	84.8 ± 11	13
	Spring 2020 (27)	89.4 ± 7.6	8.5
	Spring 2021 (28)	80 ± 14.5	18

**Table 3:** Students’ scores in the practical component of basic anatomy courses for MD and BMS programs in three different semesters. CV: Coefficient of variance is calculated as SD/Mean × 100.

Course	Semester (No. of Students)	Mean ± SD	Coefficient of Variance (%)
MD program basic anatomy course	Spring 2019 (170)	77.4 ± 15.9	20.5
	Spring 2020 (166)	85.8 ± 10	11.7
	Spring 2021 (129)	78.7 ± 17.1	21.7
Biomedical Science program basic anatomy course	Spring 2019 (33)	75.7 ± 13	17.2
	Spring 2020 (27)	87.6 ± 9.4	10.7
	Spring 2021 (28)	80.4 ± 12.1	15

## Data Availability

Not applicable.

## Supplementary Materials

The following supporting information can be downloaded at: https://www.mdpi.com/article/10.3390/ijerph192013318/s1, Table S1: Gender distribution of students included in the study over the three semesters.
