# Supplementary material for: Students’ Performance in Face-to-Face, Online, and Hybrid Methods of Teaching and Assessment in Anatomy
Source: Int J Environ Res Public Health. 2022 Oct 15;19(20):13318. doi: 10.3390/ijerph192013318 (PMC9602488; doi:10.3390/ijerph192013318)
Supplement: Supplementary file 1 [file ijerph-19-13318-s001.zip › ijerph-1940552-supplementary.pdf]

Supplementary Table S1: Gender distribution of students included in the study over the three semesters.

**MD program**	Spring 2019	Spring 2020	Spring 2021
Male students	79	81	64
Female students	91	85	65
Total number	170	166	129

**BMS program**	Spring 2019	Spring 2020	Spring 2021
Male students	14	14	14
Female students	19	13	14
Total number	33	27	28
